# Clinical pointers in *Prevotella* septic arthritis of the hip:  a case report

**DOI:** 10.1186/s13256-023-03961-7

**Published:** 2023-06-10

**Authors:** M. P. Kgagudi, M. G. Mogane, M. T. Ramokgopa, M. Jingo

**Affiliations:** Division of Orthopaedic Surgery, School of Clinical Medicine, Faculty of Health Sciences, University of the Witwatersrand, Johannesburg, South Africa

**Keywords:** Septic arthritis, Infective arthritis, *Prevotella* spp., Case report

## Abstract

**Background:**

Infective arthritis is an orthopaedic surgical emergency. *Staphylococcus aureus* remains the commonest causative bacteria across all age groups. *Prevotella* spp. as a cause of infective arthritis is extremely rare.

**Case report:**

We present our case of a 30-year-old African male patient who presented with mild signs of infective arthritis of the left hip. His risk factors were his background retroviral disease, intravenous drug abuse, and a previous episode of left hip arthrotomy which healed expectantly with intervention. The current presentation was treated with arthrotomy of the hip, fluid lavage, and skeletal traction based on our clinical findings and the rarity of the presentation was seen to be mobilising non-weight bearing with crutches, and pain-free on the left hip.

**Conclusion:**

A high index of suspicion for Prevotella Septic Arthritis (PSA) should be exercised when treating infective arthritis patients with background joint arthropathies, and intravenous drug abuse, especially in individuals with significant immunosuppression and/or recent tooth extraction. Fortunately, although rare an entity, good outcomes can be expected with early diagnosis and classic treatment principles of joint decompression and lavage as well as guided antibiotic therapy.

## Introduction

Infective arthritis is one of a few orthopaedic surgical emergencies [1]. Bacterial septic arthritis is by far the commonest form of infective arthritis [1, 2]. *Staphylococcus aureus* (SA) accounts for between 70 and 90% of cases of infective arthritis with the remainder of the cases being caused by either other gram-positive, gram-negative, mycobacteria or anaerobic organisms [1–3]. The latter micro-organisms rarely affect synovial joints, especially *Prevotella spp.* [4] and as such we present our case report of *Prevotella* septic arthritis (PSA) of the hip with particular emphasis on a clinical approach with pointers for making a diagnosis, all the way through to rehabilitation of the affected joint.

## Case report

We present a 30 years old African male who reported a 4-day history of worsening left hip pain, swelling, and inability to weight bear on the left lower limb. He gave a background history of being retro-viral disease reactive, which was uncontrolled on treatment (CD4 = 218 cells/ml, viral load = 3320 copies/ml). Off-note is that he also suffered from pulmonary tuberculosis (December 2021) which was treated successfully with no sequelae, however, he was also known to suffer from intravenous drug addiction using his arm veins for the injections. He denied any prior dental procedures but gave a history of previous left hip septic arthritis a year prior to the current presentation, he was treated with surgical joint decompression and antibiotics, and an unremarkable recovery was reported.

### Clinical findings

**Fig. 1:**
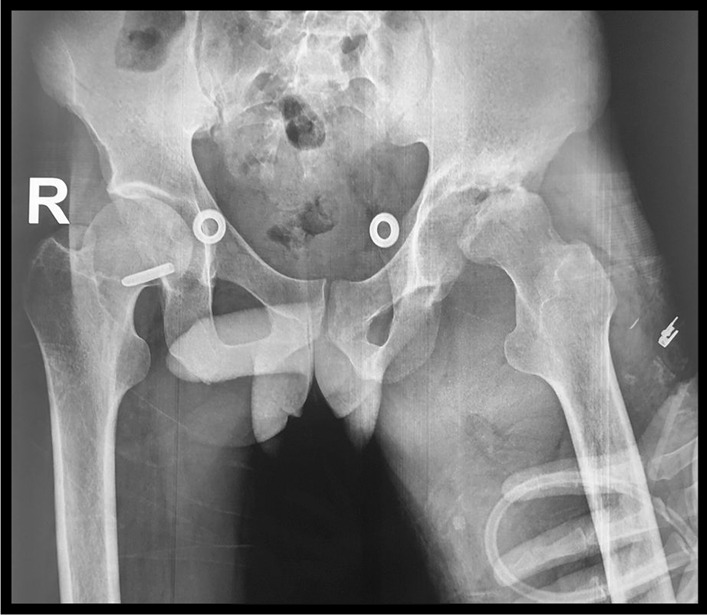
Anteroposterior radiograph showing the pelvis

On examination, the patient was generally ill-looking, vitals (BP = 119/88 mmHg, HR = 101 b/m, RR = 20 b/m, Temp = 36.4 °C), and the left hip was held flexed, abducted, externally rotated, and irritable to examination with marked tenderness. On radiographs, see Fig. 1 the left hip radiograph confirmed the clinical posture of the left hip with the destruction of the femoral head and a widening of the joint space with superolateral subluxation of the femoral head. Laboratory infective marker workup was in keeping with an infective process by raised septic markers (Erythrocyte Sedimentation Rate (ESR) = 113 mm/hour, C- Reactive protein (CRP) = 50 mg/L, PLTs = 532 × 10^9^/L), however, the White Cell Count (WCC) and renal function were normal.

### Therapeutic intervention

**Fig. 2:**
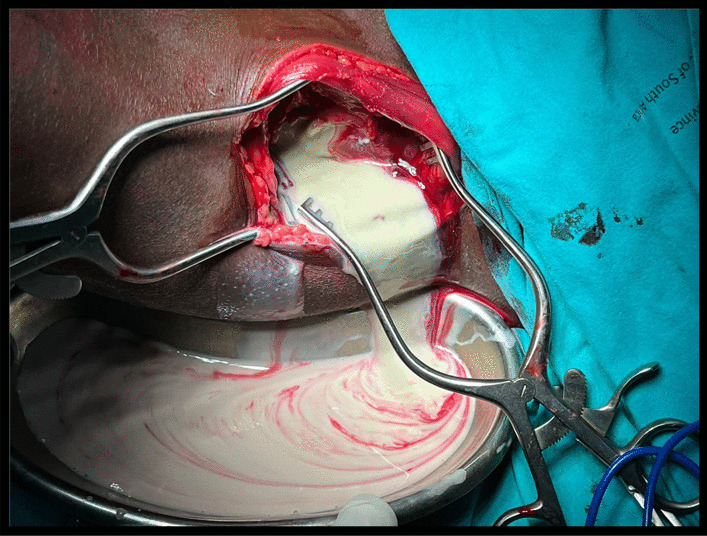
Intraoperative drainage of the left hip

**Fig. 3:**
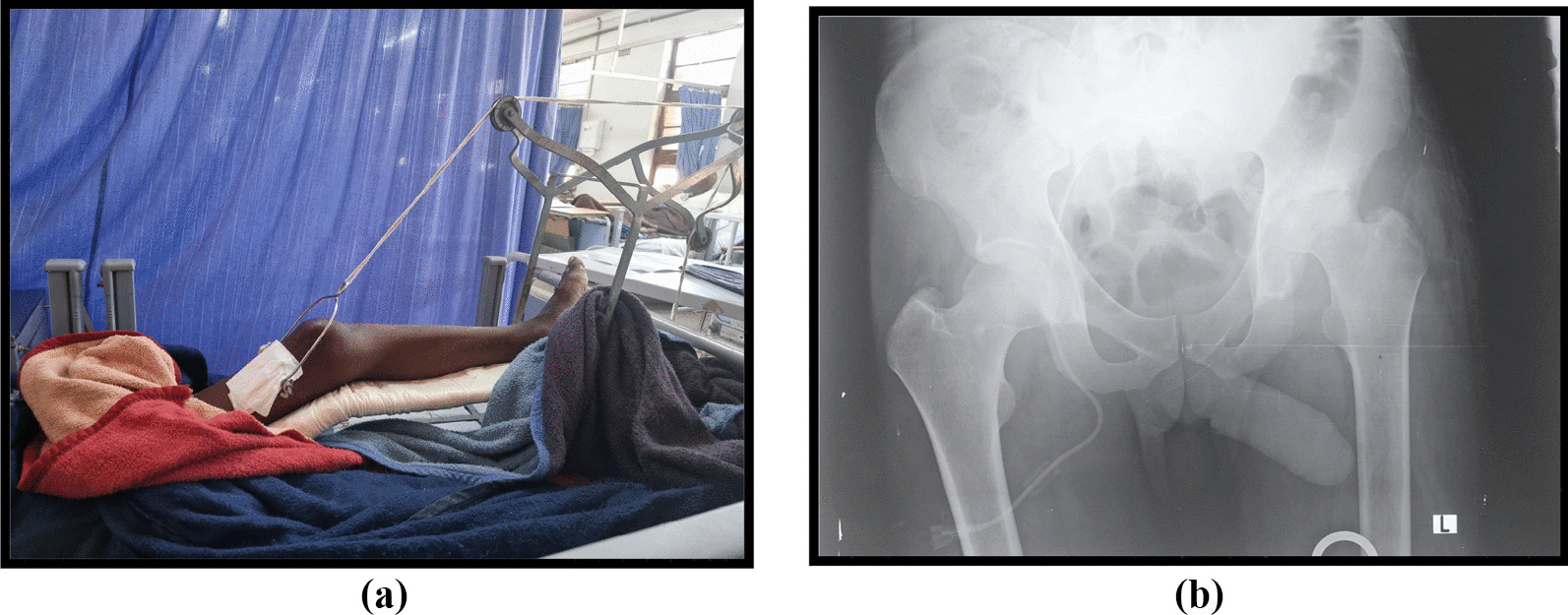
**a** 4 weeks on skeletal traction. **b** post-traction radiograph.

The patient underwent emergent hip arthrotomy, see Fig. 2. with copious yellowish pus evacuated from the hip. The hip also received extensive fluid lavage and a Porto-vac drain was left in-situ for continuous post-operative drainage in the ward. The microscopy results surprisingly revealed *Prevotella* as an infective micro-organism. The patient received intravenous antibiotics (Metronidazole 500 mg iv. ter die sumendum/three times daily (TDS), in our case) for 4 weeks and trans-femoral skeletal traction with Brown’s frame as shown in Fig. 3a which, aided in repositioning the femoral head within the acetabulum as shown in a radiograph (Fig. 3b) done at 4 weeks post-traction.

## Discussion

*Staphylococcus aureus* is still by far the commonest cause of septic arthritis [1, 2]. A patient’s age group and clinical condition usually predisposed one to infective bacteria outside of usual cases due to SA [1–3]. Rarer causes of septic arthritis include *Prevotella* species [4–6, 8]. The literature reports these micro-organisms to be isolated in only a handful of cases [4–9]. And as such, there is no level 1 evidence for diagnosis, treatment, and eventual outcomes for PSA. Our case of discussion was a young male who fits the profile for PSA as per his risk factors [4]. Shalman et al. reported the condition to affect individuals in the 5th and 6th decades but it can also be expected in younger patients suffering from medical co-morbidities and risk factors, as was the case in our patient [4–8]. On history, he had a prior surgical history of the same (LEFT) hip for a previous infective arthritis that was treated and had healed uneventfully. Naseir et al. also reported *Prevotella* septic arthritis in a joint with previous surgery. However, Shalman et.al and others reported the infection in surgically naive joints [5, 9]. Recent dental surgery has also been associated with PSA following dental tooth extraction [6, 7]. PSA post-dental surgery can develop as early as 48 hours post-tooth extraction especially in elderly patients [6]. Usually in cases that follow post-dental work there is an underlying arthropathy of sorts [7]. Joint inflammatory arthritides have always been noted to be risk factors for the development of infective arthritis on the whole [8–11].

Clinically PSA presents with the classic signs of infective arthritis with pain, swelling, warmth, and loss of function of the involved joint, however usually with an associated draining sinus [4, 5]. The picture can be easily confused with that of subacute and even chronic infective arthritis like the one seen in tuberculosis of the joints. Ironically, radiological changes with PSA are similar to those of chronic infective arthritis. Our case presented with an increased joint space and an effusion. Surgical drainage usually reveals a yellowish-to-greenish collection of pus [4]. Microscopy revealed a small gram-negative rod on Haematoxylin and Eosin staining previously referred to as Bacteroides species. Fortunately, these microorganisms are usually sensitive to antibiotics [12, 13]. However the duration of treatment is not well defined in the literature and so we adopted treatment as per the usual SA infective arthritis with the use of intravenous antibiotics for 4 weeks and an additional 2 weeks of oral antibiotics post-discharge [4, 13]. Metronidazole is the gold standard of treatment with clindamycin being the only alternative [13]. Traction was applied for the 1st four weeks with the plan to have the joint heal in an acceptable arthrodesis position of hip flexion at 15 degrees. Arthrodesis was preferred in our case since there was established joint destruction at presentation and the patient was not an ideal candidate for arthroplasty replacement due to his age and co-morbidities. At the last follow-up, the patient was seen to be mobilising non-weight bearing with crutches, and pain-free on the left hip.

## Conclusion

PSA is an uncommon cause of a common orthopaedic emergency. A high index of suspicion should exist when treating septic arthritis patients presenting with a background of general inflammatory arthritis, and/or previous total joint replacement, especially in individuals with significant immunosuppression, intravenous drug abuse, and/or recent tooth extraction. Fortunately, although rare an entity, good outcomes can be expected with early diagnosis and classic treatment principles of joint decompression and lavage as well as guided antibiotic therapy.

## Data Availability

All relevant data pertaining to the case is available for perusal by reviewers and Editor-in-Chief.
